# Relationship between Retinal Nerve Fiber Layer Thickness and Hemoglobin Present in the Optic Nerve Head in Glaucoma

**DOI:** 10.1155/2017/2340236

**Published:** 2017-06-04

**Authors:** Marta Gonzalez-Hernandez, Jose Sigut Saavedra, Manuel Gonzalez de la Rosa

**Affiliations:** ^1^Department of Ophthalmology, Hospital Universitario de Canarias, La Laguna, Spain; ^2^Department of Systems Engineering, University of La Laguna, La Laguna, Spain; ^3^Department of Ophthalmology, University of La Laguna, La Laguna, Spain

## Abstract

**Purpose:**

To observe the relationship between topographic hemoglobin levels in the optic nerve head (ONH), the rim thickness (BMO-MRW), and retinal nerve fiber layer (RNFL) thickness.

**Methods:**

96 normal eyes and 82 glaucomas were examined using TOP strategy (Octopus 300 perimeter), SPECTRALIS OCT, and Laguna ONhE program which estimates hemoglobin from conventional color photographs (Horus Scope DEC 200 fundus camera).

**Results:**

The correlation between Laguna ONhE glaucoma discriminant function (GDF) and SPECTRALIS BMO-MRW was *R* = 0.81 (*P* < 0.0001), similar to that between the BMO-MRW and BMO-RNFL thicknesses (*R* = 0.85, *P* < 0.0001) (*P* = 0.227 between both *R* values). GDF correlated well with RNFL thicknesses in the 360 degrees around the nerve, similar to mean perimetric sensitivity (MS) and BMO-MRW. The amount of hemoglobin in the nasal and temporal sectors showed low correlation with superior and inferior RNFL thicknesses. The superotemporal and inferotemporal sectors located on the vertical diameter of the disk showed good intercorrelation but without a clear RNFL topographic relationship.

**Conclusion:**

GDF showed high correlation with RNFL thickness. Except in the nasal and temporal sectors, ONH hemoglobin correlated well with RNFL thickness.

## 1. Introduction

One of the main factors that have recently been related to the optic nerve glaucomatous lesion is the vascular component [1]. Until very recently, the most accessible method of study was flow, analyzed by echo Doppler in nearby vessels [2]. Subsequently, several attempts have been made to measure oxygen concentration in the nerve itself [3] and more recently, some methods to measure vascular structure and flow through OCT angiography have been developed [4] and the amount of form elements present in the blood using laser Doppler [5] or speckle [6].

Our group has proposed a simple method to topographically measure the amount of hemoglobin in the blood from conventional color images of the optic nerve head (ONH) [7], especially noting the differences between its red and green components. The main obstacle in achieving this is that the chromatic result of the analysis of the red-green-blue (RGB) components of conventional photographic images does not depend exclusively on the absorption of hemoglobin but also on the spectral composition of the illumination light, the absorption of the lens, and the spectral response of the detector used. The essential idea that has allowed the Laguna ONhE program to resolve this dilemma is to choose a known chromatic pattern within the eye to compensate for all these variables.

**Figure 1:**
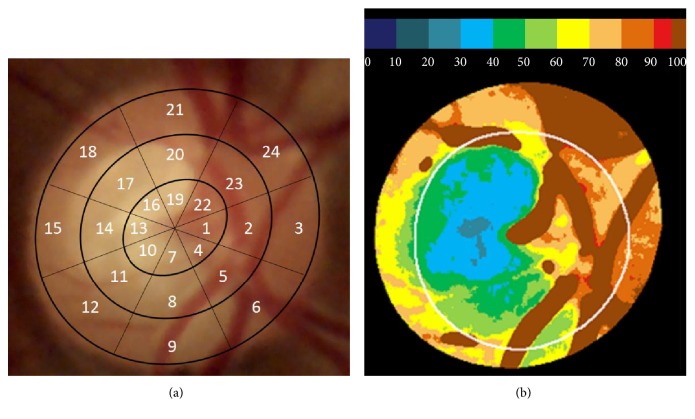
(a) A photographic image of the optic nerve head, divided into 24 regular sectors around its centroid, used by the Laguna ONhE program (3 nasal and 15 temporal). (b) The relative percentage amounts of hemoglobin in the optic nerve head tissue compared to that of the vessels, which is taken as the reference value (100%) and the estimated position of the cup.

The relative amount of hemoglobin present in the tissue of the optic nerve head (ONH) is estimated as follows: a central point in the ONH is manually identified as a provisional centroid, to facilitate the identification of its borders by automatic imaging analysis procedures, which take into account previous experiences in which photographic images were adjusted with delimitations performed using OCT. The result can be refined by the user if deemed necessary. Then, the real centroid of the delimitation is calculated, which is divided regularly by four diameters, and, in turn, divided into 6 segments by lines parallel to the contour, finally leaving the ONH divided into 24 sectors (Figure 1(a)).

Next, automatic segmentation of the image is performed to separate the central vessels of the retina from the ONH tissue. In the set of pixels corresponding to the vessels, a mathematical operation is performed, using the chromatic components red (R) and green (G), since hemoglobin preferably absorbs green and reflects red.

The difference between the two components is obtained using the formula (R − G)/R, which has been experimentally proven to provide a linear result with respect to the amount of hemoglobin present [7]. The same operation is performed in each region of the ONH tissue, and the local result is shown as a percentage of the reference value obtained in the vessels (Figure 1(b)).

The distribution of hemoglobin in these sectors is used to estimate the vertical cup-to-disc ratio. The usefulness of this estimate has been verified by our group and independent investigators [8]. The program also provides an overall glaucoma discriminant function (GDF) index, among others.

After a first multicenter study demonstrating the usefulness of the procedure, we analyzed its reproducibility [9], and it was then applied to ocular hypertensives [10]. New studies have allowed separating the rim from cup information, analyzing images obtained with a stereoscopic camera [11], and also associating two-dimensional photographic images with optical disk and cup delimitations provided by OCT [12]. Among the conclusions of these studies, two stand out: first, hemoglobin concentrations are better correlated with functional defects of glaucoma than the corresponding areas of the neuroretinal rim or RNFL thicknesses. Second, in many patients, hemoglobin concentrations in the residual neuroretinal rim are lower than normal values, suggesting that the process of nerve damage may continue in these cases.

What we do not have enough information about at the present time is whether the reduction of perfusion is the cause or consequence of nerve fiber atrophy or whether both factors coincide. To contribute to the knowledge on this aspect, the present work focused on analyzing the dependence relationships between the different sectors of the ONH with the optic nerve rim (BMO-MRW) and the topography of the RNFL thicknesses studied.

## 2. Material and Methods

In this prospective cross-sectional study, 96 eyes from 96 normal subjects (“control” group) and 82 eyes from 82 subjects with confirmed or suspected open-angle glaucoma (“glaucoma” group) seen at the Hospital Universitario de Canarias were examined. We attempted to ensure that the sample of glaucomas was representative of all the stages of the disease. The data obtained by each instrument were collected by a single investigator.

This study protocol adhered to the principles of the 1964 Declaration of Helsinki and was approved by the Research Ethics Committee of the Hospital Universitario de Canarias. Participants were informed about the study objectives and the tests that were to be performed and expressed their agreement with them.

The subjects were required to have a corrected visual acuity (BCVA) of 20/40 or better, a refractive error of less than 5 diopters of spherical equivalent or 2 diopters of astigmatism, and an open angle in the anterior chamber.

Patients in the “glaucoma” group had characteristic optic nerve defects or suspected disease data, such as intraocular pressure (IOP) greater than 21 mmHg, associated with a family history of glaucoma, a suggestive aspect of ONH, or a borderline condition in the visual field, for example, a mean defect greater than 2 dB or depressed points in the defect curve. Subjects with IOP greater than 25 mmHg or pressures between 21 and 25 mmHg associated with a corneal thickness (ECC) of less than 500 *μ*m were also included, regardless of other types of signs or symptoms.

The presence of cataracts was not considered an a priori exclusion criterion, provided that visual acuity was not reduced below 20/40. No special importance was attached to equating the samples by age, since previous studies have shown no significant changes with age in the hemoglobin values of the ONH in normal subjects [11].

Patients included in the “control” group did not have any significant ocular pathology in the eye recruited for the study, had an intraocular pressure lower than 21 mmHg, and had no family history of glaucoma. These subjects were not selected by the glaucoma section of our hospital but rather they comprised hospital staff, patients from other sections without intraocular pathology, or family members of patients. All subjects had previous perimetric experience, and none had any systemic disease that could affect vision.

All patients underwent a complete ophthalmologic examination and a perimetric study using program 32 and TOP strategy of the Octopus 300 perimeter (Haag-Streit AG, Bern, Switzerland) [13].

In all cases, two images of the ONH were obtained using the Horus Scope DEC 200 handheld camera (MiiS, Taiwan), which were analyzed with the Laguna ONhE version 4.0 (INSOFT SL, Spain), averaging the results. A detailed description of the use of the program has been published [14].

An examination with the Glaucoma Module Premium Edition (GMPE) of the SPECTRALIS OCT (Heidelberg Engineering, Germany) was also performed. The version we used allows the export, using a module called RNFL Export, of the 768 values of RNFL thickness obtained in the 360-degree circumference with respect to the center of the nerve to be calculated with respect to the ends of Bruch's membrane.

The limits of Bruch's membrane were not taken into account to delimit the edges of the ONH when using the Laguna ONhE program. However, the automatic edge identification algorithms used by this program take into account the experience accumulated in other studies in which the photographic images were adjusted with the ONH edges delimited by OCT [12].

## 3. Statistical Analysis

All statistical analyses were performed using MedCalc (version 7.3, software MedCalc (Mariakerke, Belgium)) and Excel 2016 (Microsoft, Redmond, USA). For the development of the Laguna ONhE program, the blue, green, and red components were evaluated using the MATLAB image analysis program (The MathWorks Inc., Natick, MA) and its toolbox for image processing.

After ensuring a normal distribution of the variables, Pearson correlations were also calculated between the indices provided by the perimeter and the Laguna ONhE program, as well as the indices and thicknesses provided by SPECTRALIS OCT.

## 4. Results

Demographic and clinical characteristics of the two groups studied are summarized in Table 1.

In the control group, the changes in hemoglobin with age were minimal and not statistically significant (0.033% per year), while BMO-MRW was reduced by 0.83 *μ*m per year and BMO-RNFLT by about 0.28 *μ*m per year.

**Figure 2:**
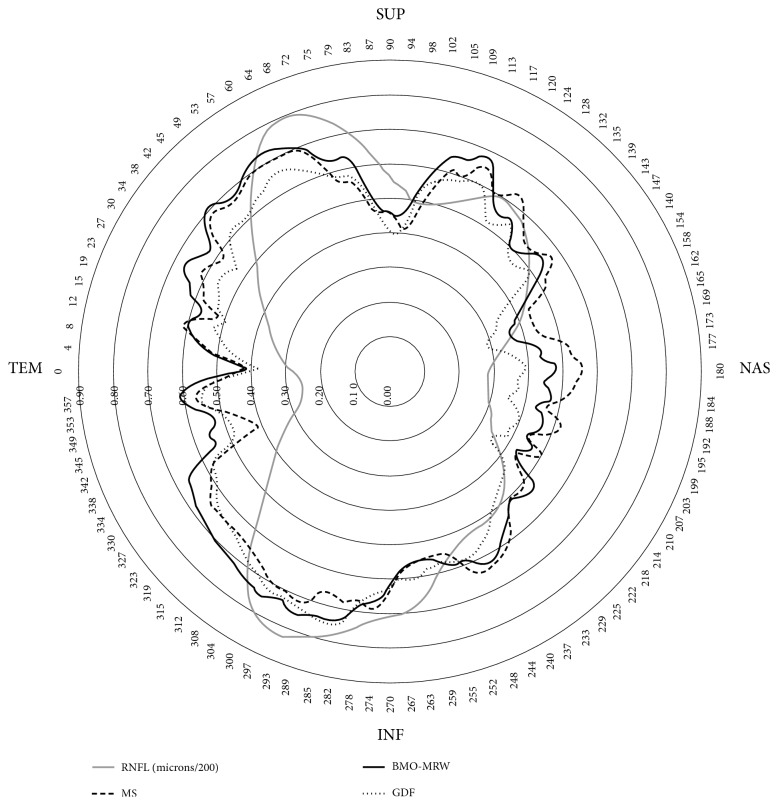
The black lines represent the correlation coefficients (the greater the coefficients, the greater the radius) of perimetric mean sensitivity (MS), SPECTRALIS OCT BMO-MRW index, and the Laguna ONhE GDF index, with respect to the 768 RNFL thicknesses measured in 360 degrees. In gray, the RNFL thickness of normal control subjects (microns/200) has been superimposed.

In Figure 2, the average RNFL thickness in the 96 eyes of the normal control sample is shown in gray. It shows that the RNFL thickness is higher in the upper and lower regions of the ONH than in the nasal and temporal regions. The correlation coefficients have a similar distribution in the three indexes that are represented: perimetric mean sensitivity (MS), fiber layer thickness measured perpendicular to the internal limit by SPECTRALIS OCT (BMO-MRW), and the glaucoma discriminant function (GDF) of the Laguna ONhE program. The coefficients are higher with respect to the upper and lower thicknesses, lower in the nasal region, and minimal in the temporal region. The mean defect (MD) of the perimetries in the upper regions was 8.8 dB (SD = 9.1) and that of the lower regions was 6.9 dB (SD = 7.9) (*P* = 0.005).

**Figure 3:**
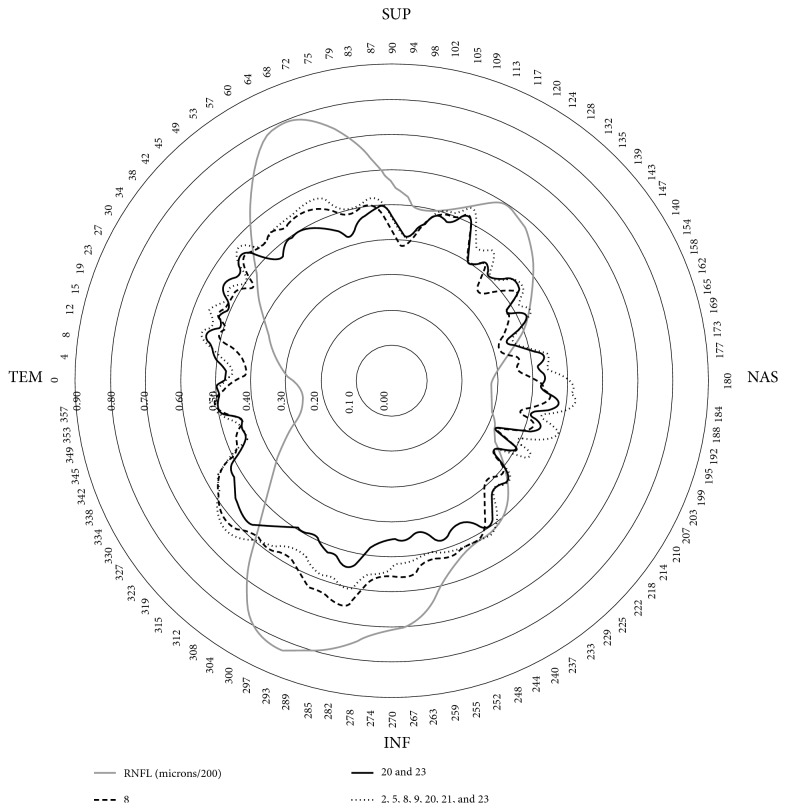
The black lines represent the correlation coefficients of the relative amount of hemoglobin in sector 8 of the optic nerve head, the average in sectors 20 and 23, and the average in sectors 2, 5, 8, 9, 20, 21, and 23, measured by the Laguna ONhE program, with respect to the 768 RNFL thicknesses. The thickness of the RNFL (microns/200) in gray has been superimposed on the image.

Figure 3 shows that the amount of hemoglobin present in sector 8 of the ONH (lower) and the average of sectors 20 and 23 had a very similar relation to the upper and lower RNFL thicknesses, as well as the average hemoglobin in various sectors located in the centrotemporal and upper and lower regions (2, 5, 8, 9, 20, 31, and 23). In all cases, the relationship was somewhat greater with the lower than with the upper thicknesses.

**Figure 4:**
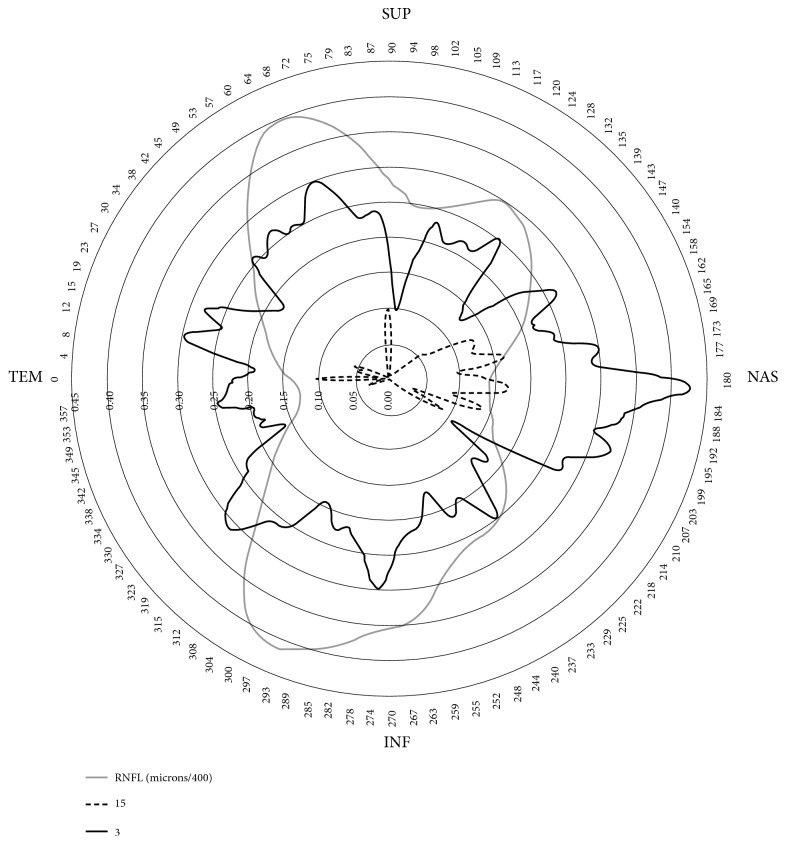
The black lines represent the correlation coefficients of the relative amount of hemoglobin in sectors 15 and 18 with respect to the 768 thicknesses of the nerve fiber layer. Some minimally negative correlations have been omitted. The thickness of the fibers (microns/400) in gray has been superimposed on the image. Note that, in this case, only coefficients less than 0.45 are represented.

Figure 4 shows that the amount of hemoglobin present in the temporal and nasal sectors of the optic nerve head has little relation with the thickness of the fiber layer, especially in sector 15 through which the maculopapillary bundle passes. In any case, there is only a modest correlation with the nasal and temporal thicknesses.

**Figure 5:**
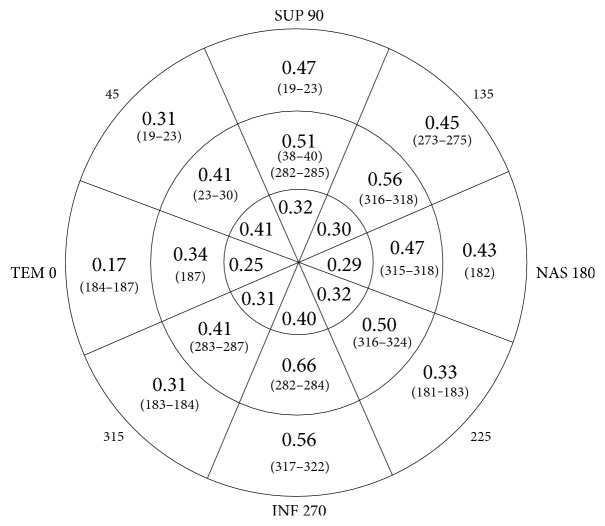
Best correlation coefficients obtained in each sector of the optic nerve head and thicknesses specified by its angular position (degrees) of the circle of fiber thickness where they were obtained (below the correlations, in brackets).

Figure 5 shows the maximum correlation values obtained in each of the 24 sectors into which the Laguna ONhE program divides the ONH. The correlations are greater in the intermediate zones, where the cup progresses and the neuroretinal rim diminishes. They reach maximum values in sectors 8, 20, and 23 but without great differences in relation to the upper or lower thicknesses. For example, the upper sector 20 achieves a maximum and identical correlation with the lower thicknesses corresponding to 282–285 degrees and with the upper thicknesses corresponding to 38–40 degrees. The central sectors have been given less interest, partly because they are too small, and also because they are often largely occupied by vessels, leaving little tissue available for measurement.

**Figure 6:**
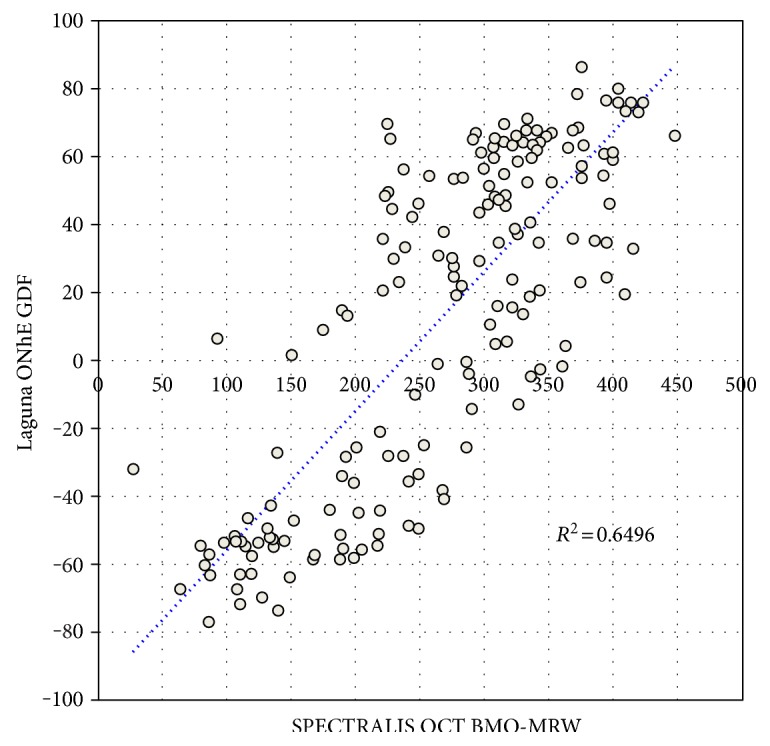
Scattergram of the BMO-MRW thickness that SPECTRALIS OCT measures in the neuroretinal rim, compared to the value of the glaucoma discriminant function (GDF) of the Laguna ONhE program.

As can be seen in Figure 6, the GDF index of Laguna ONhE, which takes into account the distribution of hemoglobin in the 24 sectors, and especially the slope of its concentrations in the vertical diameter from the periphery to the center, presents very high correlation with the mean thickness of the neuroretinal rim measured by SPECTRALIS OCT (*R* = 0.81, *P* < 0.0001).

**Figure 7:**
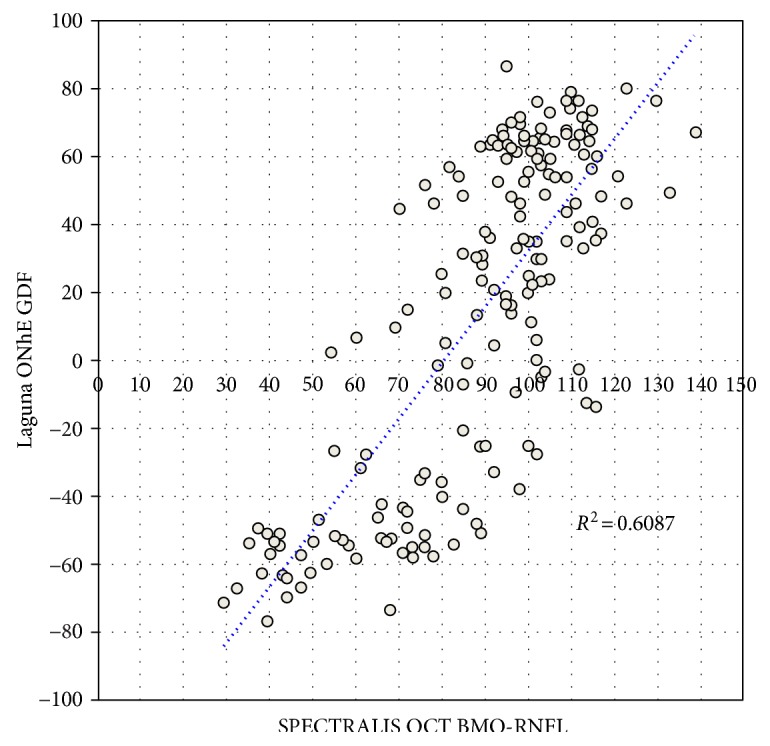
Scattergram of the BMO-RNFL thickness of the nerve fiber layer measured by SPECTRALIS OCT, compared to the value of the glaucoma discriminant function (GDF) of the Laguna ONhE program.

This correlation is comparable to that shown in Figure 7, where the mean thickness of the neuroretinal rim (BMO-MRW) in relation to the mean thickness of the nerve fiber layer (BMO-RNFL) is represented, both measured by the same OCT (*R* = 0.85, *p* < 0.0001), without significant differences between the magnitudes of the two correlations (*P* = 0.227).

The correlation between GDF and BMO-RNFL (*R* = 0.78, *P* < 0.0001) was also very high, at the limit of statistical significance with respect to the comparison of BMO-MRW versus BMO-RNFL (*P* = 0.049).

## 5. Discussion

As each ganglion cell captures information from a specific region of the visual field, numerous studies have focused on comparing the sensitivity of each region of the visual field with the corresponding anatomical sectors, whether the neuroretinal rim or the retinal nerve fiber layer (RNFL). There are significant differences, not only according to the instruments used, but also when the same instruments are used. For example, with HRT, few authors have obtained maximum correlations between 0.52 [15] and 0.77 [16], using GDF between −0.35 [17] and 0.78 [18] and using OCT between 0.51 [19] and 0.85 [20].

These differences seem to be due to multiple causes, including limitations of the particular technique used [21], interindividual variability [22], or the fact that the relationship between morphological and functional data is not linear but curvilinear [23]. Another factor described by our group is that the correlation coefficients obtained were dependent on the range of RNFL thicknesses at each point [24].

Several of these factors may serve to interpret the results of the correlations that we obtained in the present work between the amount of hemoglobin and RNFL thicknesses. One of the problems mentioned above does not seem to have any influence in this case, because we consistently observed a linear relationship between the morphological parameters and those obtained by measuring hemoglobin. On the other hand, the correlations between the sectoral presence of hemoglobin and the RNFL thickness in sectors 3 and 15 may depend mainly on anatomical variants of each subject, even to a greater extent than the stage of the disease. This interpretation would explain, for example, that the hemoglobin of the temporal area 15, infrequently affected by glaucoma, is especially related to the RNFL thicknesses on the opposite side (nasal).

The areas of the ONH which individually exhibit higher correlations with the RNFL thicknesses do so in a poorly selective manner, as seen in Figures 3 and 5. Perfusion alteration seems to be somewhat specific in this regard, although the correlations are somewhat larger with respect to the thicknesses of the inferior zone of the ONH, corresponding with the greater frequency of superior functional defects.

Finally, the range of RNFL thicknesses may influence the magnitude of some of the correlation coefficients observed. Especially, the correlations of the GDF index, like those of other global indices such as perimetric MS or BMO-MRW of SPECTRALIS OCT, tend to reproduce in their magnitude the difference in RNFL thickness in each region of the rim measured, as shown in Figure 2.

It is particularly noteworthy that the correlations between the Laguna ONhE GDF index and the mean thickness indices provided by SPECTRALIS OCT are practically as intense as those provided by these OCT indices with respect to each other.

If there is a relationship between tissue loss and hemoglobin levels, and at the same time the residual tissue of the rim shows hypoperfusion, then perfusion deficit is conditioning the loss of tissue. However, this would be only one more argument in favor of this hypothesis. The definitive argument would need demonstration, in longitudinal studies, that the tissue is lost only when it is hypoperfused and not when residual neuroretinal rim perfusion is normal.

Laguna ONhE is currently able to estimate the size and position of the cup, without needing stereoscopic or OCT information, and divides the rim into sectors that can be compared with equivalent morphological sectors. In order to analyze the results in this way, the colocation of the rim sectors with respect to OCT is very important [11, 12], but this was not our intention in the present work. In the progression of glaucoma, the cup grows at the expense of rim reduction in the intermediate zones of the ONH. If we separate the rim from the cup, it is not easy to perceive that the hemoglobin of the border zone between them influences the growth of the cup. For this reason, we have reused the regular division of the ONH in sectors, as in our first work [7].

Our view is that the ONH is relatively small, and its vascularization is not topographically specific. We have observed more ischemia in some areas than in others, but in axonal injury, other factors must intervene, such as stiffness of the lamina cribrosa and intracranial pressure.

Apart from Laguna ONhE, there are currently no other methods that correlate the presence of hemoglobin in the optic nerve head with structural or functional data, although studies have been carried out that correlate these parameters with vascular flow in this area, either using laser speckle flowgraphy (LSFG) [25–28] or OCT angiography [29].

A limitation of the work is that, while age does not seem to influence hemoglobin levels, anatomic values are slightly modified throughout life, so that it may have a discrete influence on the correlations observed. In this regard, the data obtained in the present study are similar to those reported by other authors [30–32].

## 6. Conclusions

From the results obtained in this work, we can conclude that hemoglobin concentrations in the superior and superotemporal regions as well as in the inferior and inferotemporal regions of the ONH have an evident dependent relationship with the thinning of the RNFL, but without a close topographic relationship region by region. The GDF index has a very high correlation with the mean thickness of the RNFL and the thickness of the neuroretinal rim in the ONH itself, indicating that perfusion and axonal atrophy are intimately linked to each other.

## Figures and Tables

**Table 1:** Demographic and clinical characteristics of the two groups. Differences with a *p* value of <0.05 were considered statistically significant (in bold).

	Control group (average ± SD)	Glaucoma group (average ± SD)	*P*
Number	96	82	
Gender (male/female)	33/63	47/35	0.146^∗^
Age (years)	44.26 ± 12.33	65.53 ± 10.37	**<0.001** ^∗∗^
BMO-MRW (OCT, *μ*m)	333.04 ± 49.98	188.94 ± 75.21	**<0.001** ^∗∗^
BMO-RNFL (OCT 3.5 mm circle, *μ*m)	103.14 ± 11.22	70.59 ± 21.15	**<0.001** ^∗∗^
Average mean defect (dB)	0.35 ± 1.69	7.79 ± 7.53	**<0.001** ^∗∗^
Average sLV (dB)	1.86 ± 0.65	4.46 ± 2.53	**<0.001** ^∗∗^

^∗^Chi square test. ^∗∗^Student's *t*-test. OCT: optical coherence tomography; dB: decibels; sLV: square root of the loss variance.
